# Terahertz imaging for non-destructive porosity measurements of carbonate rocks

**DOI:** 10.1038/s41598-022-22535-z

**Published:** 2022-10-26

**Authors:** Jacob Bouchard, Shannon L. Eichmann, Hooisweng Ow, Martin Poitzsch, Douglas T. Petkie

**Affiliations:** 1 Department of Physics, Worcester Polytechnic Institute, Worcester, MA USA; 2 Aramco Americas: Aramco Research Center–Houston, Houston, TX USA; 3 Aramco Americas: Aramco Research Center–Boston, Cambridge, MA USA

**Keywords:** Imaging and sensing, Characterization and analytical techniques

## Abstract

Within the petrochemical industry, accurate measurement of microporosity and its distribution within core samples, particularly those from carbonate reservoirs, has garnered intense interest because studies have suggested that following primary and secondary depletion, a majority of the residual and bypassed oil may reside in these porosities. Ideally, the microporosity and its distribution would be determined accurately, quickly, and efficiently. Imaging techniques are commonly used to characterize the porosity and pores but accurate microporosity characterization can be challenging due to resolution and scale limitations. To this end, this study describes the development and verification of a novel method to characterize microporosity in carbonate rocks using terahertz time-domain spectroscopy and exploiting the high signal absorption due to water at these high frequencies. This new method is able to measure microporosity and the results agree well with other bulk measurements and produce microporosity maps which is not possible with many bulk characterization or imaging methods. These microporosity maps show the spatial variation of micropores within a sample and offers insights into the heterogeneity of reservoir materials.

## Introduction

Fluids in conventional hydrocarbon reservoirs are stored in and transported through micro- to nano-scale pores in the siliciclastic and/or carbonate minerals. Porosities in sandstone reservoirs typically have narrow size and homogenous distribution throughout the rock matrix, whereas carbonate reservoirs, which account for up to 60% of the global petroleum reserves, are characterised by heterogeneity in pore type, size and distribution^1,2^. Production and reservoir management decisions are guided by critical information obtained from a suite of interrogation methods that illuminate the characteristics of the reservoirs with resolution between nanometers to kilometers length scale. The amount of microporosity (percentage of pores < 1 µm diameter) in carbonate reservoirs and its distribution within the rock matrix have garnered intense interest because studies have suggested that following primary and secondary depletion, majority of the residual and bypassed oil may reside in these porosities^3–7^. While the petroliferous locations of the reservoir are elucidated by seismic surveys during exploration, it is the crucial information from core analysis of recovered subsurface rocks at the millimeter to nanometer length scale that affirms the production potentials of the well site. Judicious laboratory analyses of subsurface rocks allow geologists and engineers to gain an understanding of how the rock properties change with depth and in turn use this techno-economical understanding to first safely drill and complete an oil well, and then efficiently and economically produce from it^8–12^. When analyzing core materials the petrophysical features of interest are the permeability, wettability, porosity, and pore size distribution^1,10,13,14^. Typical methods of measuring porosity include mercury intrusion, optical and scanning electron microscopy, computed tomography, and gas porosimetry^1,12–20^. Accurate, non-imaging methods may be destructive or make use of hazardous materials to provide bulk measurements of porosity. Imaging methods, however, can be limited by a trade-off between field-of-view (FOV) and resolution to produce porosity maps. Thus, other imaging or spectroscopic methods to non-destructively map the microporosity distribution in carbonate rocks can provide key insight to the rock structure.

Common imaging methods for evaluation of porosity and pore size distribution in conventional reservoir rocks include X-Ray CT, optical microscopy, and scanning electron microscopy (SEM). The 100 s to 10 s of microns/pixel resolution of whole core and core plugs as provided by CT and micro-CT provide 3D images is insufficient to fully map microporosity distributions in carbonate rocks, since a significant portion of the porosity is with diameters less than 1 µm. To improve resolution, in some examples heavy ion-dope fluids imbibed into rocks have been used to track fluid mobility due to forced displacement and diffusion within the otherwise unresolvable porosity in x-ray CT imaging^21,22^. Petrograph using optical and fluorescence microscopy, with resolutions ranging from a few hundred nanometers up to 10 s of microns has proven to be quite versatile in providing information about mineralogy, depositional environment, and porosity and pore size distribution which are used to infer reservoir quality^5,23–26^. Scanning electron microscopy (SEM) provides the highest resolution of these imaging techniques, down to a few nanometers/pixel, with which both 2D and 3D rock images can be generated, and porosity and pore size distribution measured using image processing methods. While large tiled 2D SEM (pores larger than ~ 5 nm) and laser scanning confocal microscopy (pores larger than ~ 200 nm) images can be collaged at high resolution to preserve the ability to image an entire plug face, the processing of these images, which are typically very large, is challenging, leading to slow turnaround time between data collection and quantification.

Terahertz time-domain spectroscopy (THz-TDS) which operates over the 100 GHz (~ 3 mm) to 10 THz (~ 30 μm) bandwidth has seen much development in the past 30 years^27–30^. The spectrometer produces a 0.5 ps wide THz pulse which transmits through the sample. Based on the material properties of the sample, different proportions of the pulse will be scattered, reflected, absorbed, or transmitted. The transmitted pulse is detected by the spectrometer, but one can find the absorbed and scattered frequencies in the spectrum as well by performing a Fourier transform on the detected time-dependent signal. This method has found applications in the investigation of chemicals^31^, restoration of artwork^32,33^, water content of tissues and plants^34–36^, detection of subsurface defects^37^, and imaging for security applications^38^. Previous work has laid the foundation on using THz-TDS﻿ to measure bulk porosity in rocks^39,40^. More specifically, it has been demonstrated that the porosity of carbonates can be quantified by capitalizing on the strong water absorption in the THz spectrum and observing its change during dehydration^41^. This prior study, however, did not demonstrate mapping of the microporosity distribution in carbonate rocks which is of great interest for reservoir characterization.

As the field of THz spectroscopy matures, turnkey benchtop setups that obviate the need to assemble most of the optics have been developed. Fully featured software packages with these off-the-self systems also greatly simplify the data collecting process, further lowering the barrier for the integration of THz spectroscopy as a porosity measurements and mapping technique into a core analysis workflow. In this paper we demonstrate a method of using ﻿THz-TDS of carbonate rocks at different water saturation states to generate 2D microporosity maps. The 2D maps show spatial variations in the microporosity within each sample and allow comparisons of the microporosity distributions of various samples that cannot otherwise be resolved by most imaging techniques. When the THz-TDS microporosity map data are complemented with mass balance which provides bulk porosity, the relative amount of microporosity in each sample can be estimated.

## Results

### THz maps

**Figure 1:**
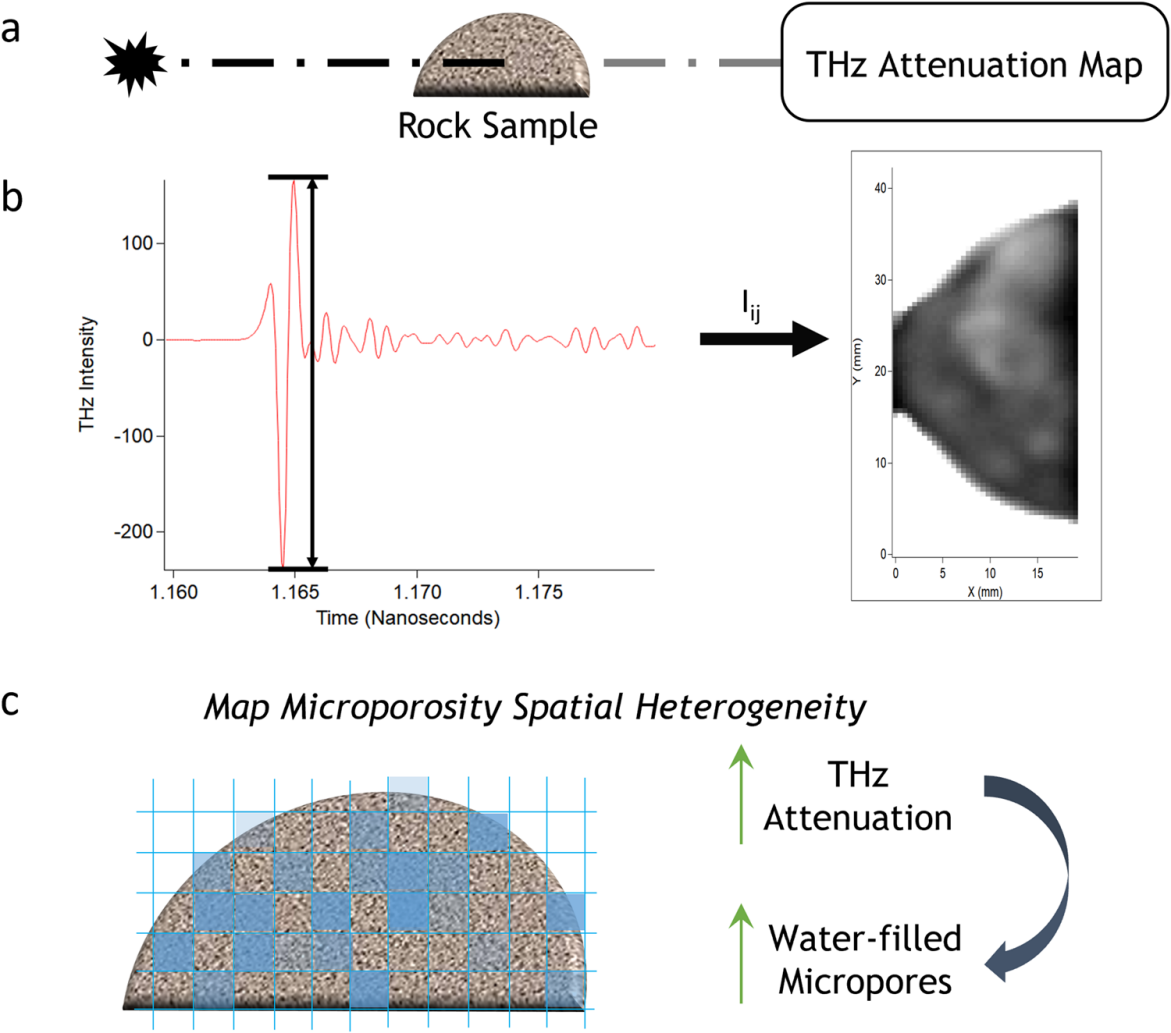
Schematic of the measurement scheme and conceptualized results. (**a**) The Terahertz (THz) beam of *I*_*0*_ passing through the rock sample and the reduced intensity, *I*_*ij*_, due to attenuation that produced the attenuation map. (**b**) The THz-TDS pulse (red) and the marked peak-to-peak amplitude (black) used to generate the attenuation map where the darkest pixels are the most attenuating and the brightest the least. (**c**) Conceptualized results showing that with increasing water-filled pores the THz attenuation increases.

Figure 1 shows the measurement scheme for collecting the THz attenuation maps of rock samples. The pre-cleaned carbonate rock samples, either saturated, centrifuged, or dry, are placed in the THz beam of intensity *I*_*0*_. The sample is then rastered through the beam at 0.5 mm steps. At each ij position, the intensity, *I*_*ij*_, of the THz beam after passing through the sample is recorded to produce an attenuation map (Fig. 1a). Figure 1b shows a representative THz pulse, the peak-to-peak amplitude of which is used to generate the normalized attenuation map, where the change in the attenuation is related to water saturation and varying elemental composition across the rock surface. Figure 1c shows conceptually how the changes in the attenuation at each 0.5 mm pixel might vary in a centrifuged sample. The pixels with the largest attenuation would contain the most water-filled microporosity with pore diameter (*d*_*p*_) less than the selected capillary pressure cut-off.

**Figure 2:**
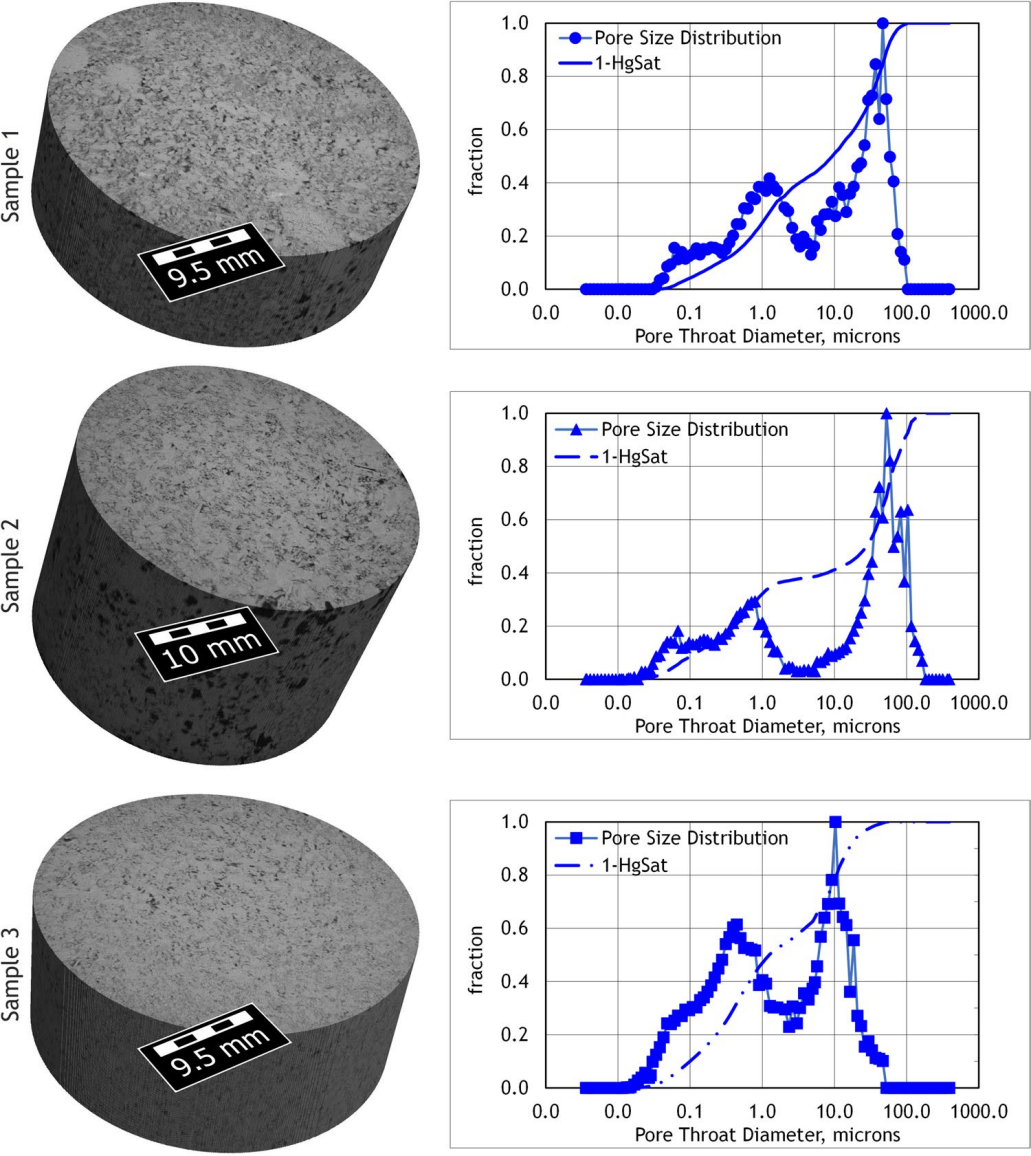
X-Ray CT Scan Renderings and MICP Pore Size Distributions. (left) CT scan renderings of the portions of each sample used for MICP analysis. Voxel size is 50 µm × 50 µm × 50 µm. The black to dark grey voxels are large pores and the light grey voxels contain matrix minerals and unresolved pores. (right) Normalized PSD and cumulative curves from MICP.

Figure 2 shows CT scan renderings at 50 µm × 50 µm × 50 µm of the larger samples used for mercury injection capillary pressure (MICP) analysis and the normalized pore size distribution (PSD) obtained by MICP from each. Due to the resolution of this imaging method, in these images only the pores larger than the voxel size are visible. The relative amount of large to small pores of each sample is shown the normalized PSD. The cumulative curve, 1-HgSat, for each sample provides the percentage of the porosity below each specified *d*_*p*_ threshold.

**Figure 3:**
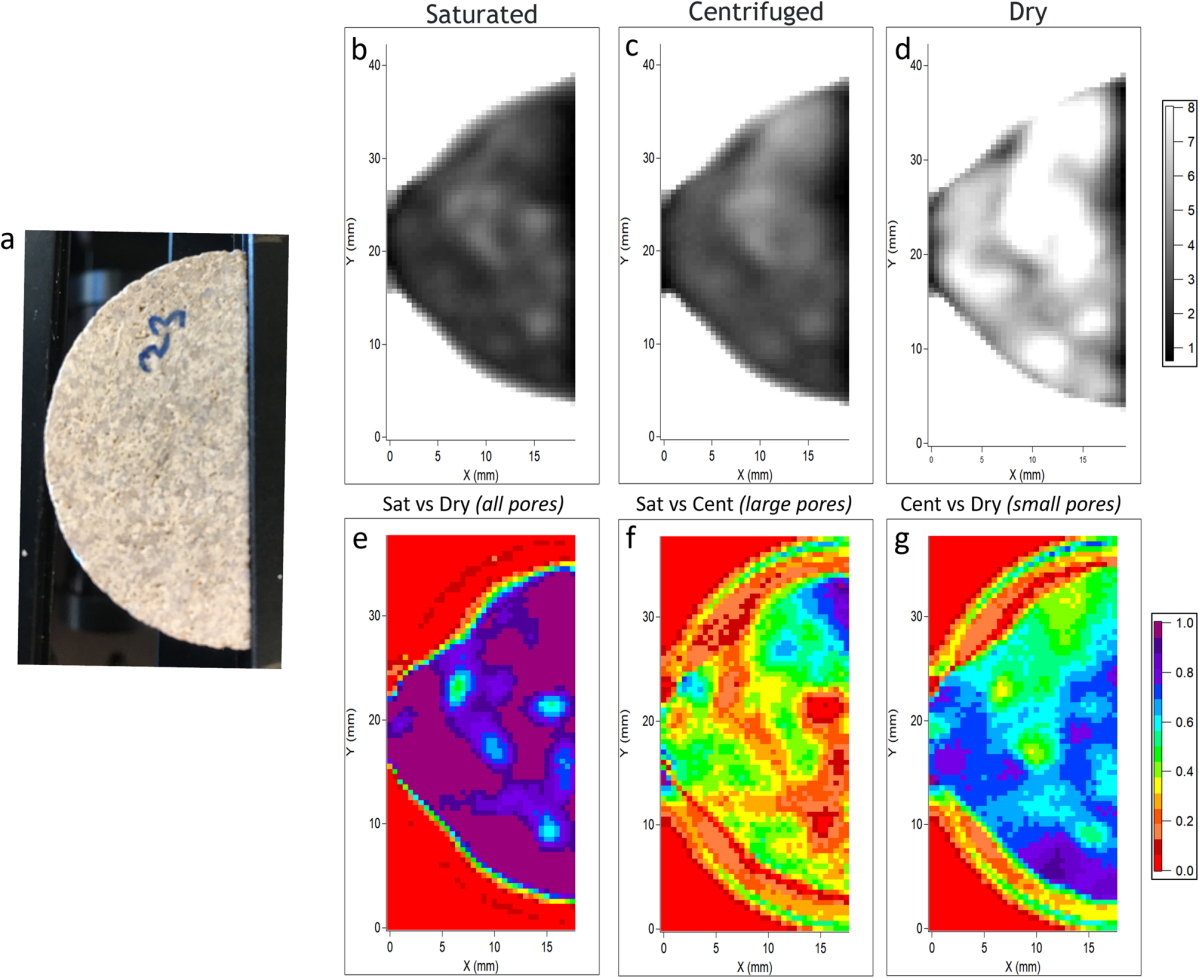
Attenuation and attenuation difference THz maps of Sample 3 (4 mm thick) as representative examples of the data for all samples. (**a**) photo, (**b**–**d**) normalized attenuation maps of the saturated, centrifuged, and dry samples, and (**e**–**g**) difference maps between the saturated and dry, saturated, and centrifuged, and centrifuged and dry attenuation maps to represent the spatial variation of the saturated pores, macropores (*d*_*p*_ > 1 µm), and micropores (*d*_*p*_ < 1 µm).

Figure 3 shows a photo of a 4 mm thick section of Sample 3, its attenuation and difference maps obtained from a pore clearing threshold of *d*_*p*_ > 1 µm. See Figs. S1 and S2 in the Supplemental Information for examples of Sample 1 and Sample 2. The color variations in the photo (Fig. 2a) are due to compositional variance in mineralogy, cementation, porosity, and surface roughness as confirmed by both the micro X-ray fluorescence (micro-XRF) elemental maps (Supplemental Information, Fig. S3) and CT reconstructions (Fig. 2). The grayscale intensity variations in the attenuation maps shown in Fig. 3b–d display changes associated with the presence of water, scattering, and to a lesser extent, the rock composition across the sample. Thus, the darkest pixels in the saturated (Fig. 3b) and centrifuged (Fig. 3c) maps mark regions of the samples that contain the most water. Accordingly, the intensity variations in the dry map (Fig. 3d) are attributed to compositional and structural variations of the sample, where the brightest regions are those that do not attenuate the THz beam. Compositional, surface topographical, and porosity differences in the three samples resulted in different baseline attenuations, which manifest primarily as variations in the scattering of the THz beam. Artifacts due to the knife edge effect, caused by the diffraction of the radiation around the sharp edge of the sample, are also visible as a high-intensity ring that bounds the sample in Fig. 3b–d. This blurring of the THz signal at the periphery of the sample leads to difficulties in demarcating the actual boundaries of the sample.

The difference maps in Fig. 3e–g show how the attenuation changed between steps in the workflow where the sample is saturated with water, centrifuged, and dried. The degree of change between the two maps is indicated by the pixel color. The difference map generated by comparing the saturated versus dry attenuation maps (Fig. 3e) shows the water-saturated regions of the sample. Similarly, comparing the saturated versus centrifuged attenuation maps (Fig. 3f), we find the regions of the sample that undergone the most loss of water as induced by the centrifugation process from pores that are larger than the cut-off (i.e., macropores). It follows that the areas of the difference map we obtain by comparing the centrifuged versus dry attenuation maps (Fig. 3g) would then highlight the regions of the sample that lost water only during the drying process, which could only be attributed to water that remains in pores smaller than the cut-off (i.e., micropores).

### Porosity and microporosity comparisons

**Figure 4:**
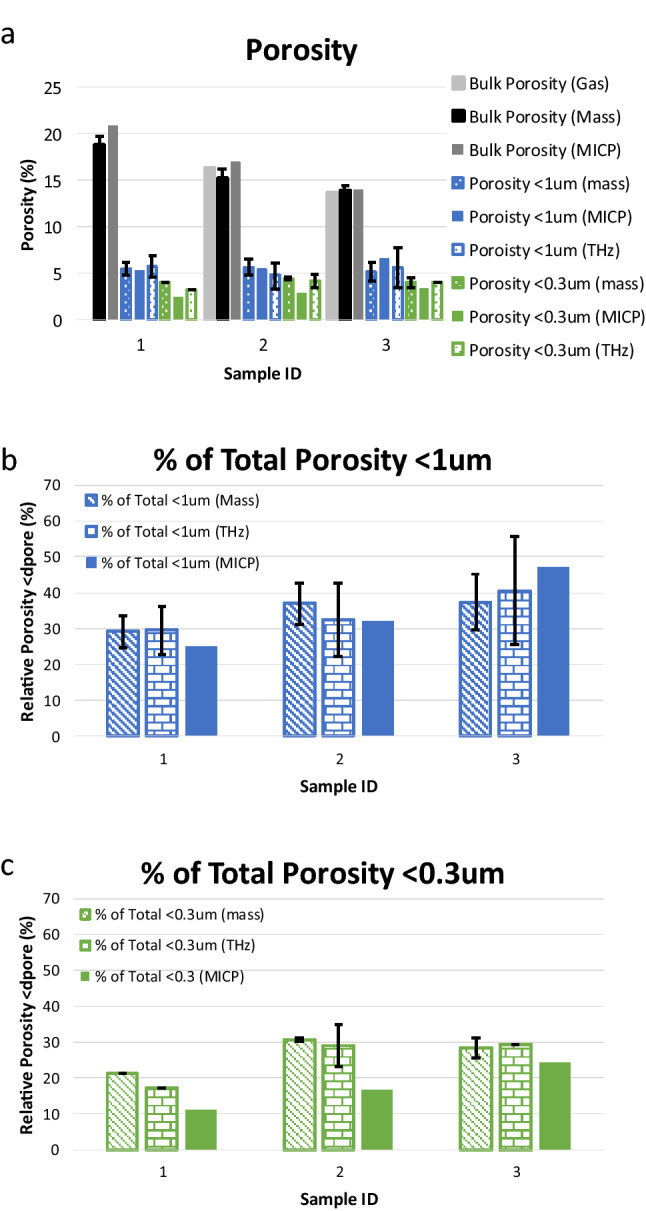
Porosity and microporosity comparisons. (**a**) Total porosity and porosity below each microporosity threshold for Samples 1–3. Total porosity from gas porosimetry (light grey) and MICP (dark grey) are measured on plug and half-plugs of each sample while the total porosity by mass (black) is the average of all measurements on the thin half-moon samples used for THz imaging. The porosity below each microporosity threshold from mass and THz is from the data collected on the half-moon samples and that from MICP is from the half-plug samples (see Fig. 3). THz and mass data are the average of data collected and the error bars represent one standard deviation. (**b**,**c**) Relative amount of the total porosity attributed to the microporosity (b: *d*_*p*_ < 1 µm and c: *d*_*p*_ < 0.3 µm).

Figure 4 compares the porosity as measured with different methods. The total porosity (Fig. 4a) was measured on the full plug by gas porosimetry (gas), on half-plugs by MICP (MICP), and on the THz samples by mass balance (mass). The porosity (macro and micro) below the *d*_*p*_ = 1 µm (blue data) and *d*_*p*_ = 0.3 µm (green data) pore clearing cut-off (Fig. 4a) was measured on the THz samples by mass (mass), from the THz maps (THz), and on half-plugs by MICP (MICP). The mass balance and THz porosity and microporosity data are the average of the measurements with the error bars of one standard deviation. Figure 4b,c show the percentage of the total porosity that is microporosity as measured with the mass balance and THz maps on the 4 mm thick rock section, compared to what was measured by MICP on half-plugs for the *d*_*p*_ = 1 µm (blue data) and *d*_*p*_ = 0.3 µm (green data) pore clearing cut-off. The mass balance and THz porosity and microporosity data are the average of the measurements with the error bars at one standard deviation.

## Discussion

Utilizing the strong attenuation of THz radiation by water, this paper demonstrates the use of THz-TDS to map pore structure heterogeneity (Fig. 3) in carbonate rocks. Three carbonate rocks with different porosity and PSD were used to elucidate the effectiveness of the method. When the samples were centrifuged to selectively expel water in pores at a selected pore size threshold, the THz-TDS maps provide a unique capability to register large FOV microporosity distributions (Fig. 3) within these bimodal pore systems. A significant amount of global oil production comes from conventional carbonate reservoirs, which make up about 60% of the conventional petroleum reservoir rocks in the world, with rocks that exhibit such heterogeneous pore characteristics^1,2^. Waterflooding processes to recovery hydrocarbon tend to bypass microporosity in the formation. Due to the significant fraction of oil-in-place in micropores and the prevalence of these pores in carbonate formations, the unswept residual oil remains a missed opportunity for overall hydrocarbon recovery^4,6,7^.

The workflow presented also demonstrates the ability to quantify the percentage of the microporosity at each pore size threshold when the data obtained from the THz system are complemented with mass balance on the samples (Fig. 4). Total porosity and microporosity obtained from the MICP technique are in good agreement with our method. It should be noted that the error bars presented in the THz and mass balance data encompass results from multiple analyses on rock sections of each sample of nominally the same thickness, as well as analyses on slices of different thicknesses. Comparisons of the dry mass before and after each saturation, centrifugation and drying run indicated that small but measurable mass loss occurred during the process. This was attributed to erosion processes and potential loss of mechanical stability of small pores and rock material along the boundaries. Nonetheless, the comparison between the porosity and relative amount of microporosity measured for *d*_*p*_ > 1 µm pore clearing threshold are in excellent agreement amongst all three methods (mass balance, THz maps, and MICP) (Fig. 4a,b). Similarly, these data measured using the mass balance and THz maps for the *d*_*p*_ > 0.3 µm threshold also agree well, albeit are consistently larger than results obtained from MICP analysis (Fig. 4a,c). This is likely due to the different sample dimensions and configurations as required by the measurement methodologies. The mass balance and THz maps are measured on the same half-moon sample slice whereas the MICP was measured on a half plug. With the heterogeneity of the samples as observed in the CT reconstructions as well as the sparsity of accessible microporosity below the *d*_*p*_ = 0.3 µm microporosity threshold, it is possible that the thin sections used for THz imaging and mass balance contain of more connected micropores upon sub-sampling. In the case of the intact half-plug used for MICP, these micropores remain less accessible.

In summary, the workflow presented in this paper is a first demonstration of using THz-TDS to map and quantify porosity in carbonate rocks which are characteristically bimodal in their pore size distribution that contains a significant amount of microporosity. In addition to the ability to map microporosity spatial variations, the excellent agreement between the relative amount of microporosity at ${d}_{p} < 1 \; \upmu {\text{m}}$, the threshold for formation evaluation in the petroleum industry, obtained by THz-TDS when compared with industry-standard MICP analysis, lends tremendous confidence for THz-TDS to be incorporated as a part of special core analysis workflow that provides valuable quantitative information on microporosity distribution in carbonate rocks.

## Methods

### Samples

Three carbonate core plug samples of 1.5″ diameter were used for this work. The Samples 2 and 3 core plugs were cut into 2 mm, 4 mm, and 6 mm thick wafers and the Sample 1 core plug was cut into 2 mm and 4 mm thick wafers which were then cut in half to produce half-moon shaped samples for THz-TDS imaging. The Sample 1 plug was shorter than the other two. As such, to allow enough materials for subsequent testing, 6 mm thick wafer was not cut from the core plug. The remaining half of each sample was used for additional characterization. Each sample is pre-cleaned to remove any pore occluding dust from the cutting process by submerging the sample in water and placing under vacuum for 2 days. The sample is then centrifuged and dried in an oven at 100 °C.

### Terahertz time domain spectroscopy (THz-TDS)

Terahertz time domain spectroscopy (THz-TDS) probes samples with short pulses of terahertz radiation. The system monitors both the amplitude and phase of the pulse which are variably affected by the properties of the sample being investigated. The resolution afforded by THz wavelengths enables the investigation and imaging of small features. More relevantly, THz radiation also interact with water molecules. Specifically, water strongly absorbs THz radiation, attenuating the interrogation pulse. Capitalizing on the attenuation of THz radiation by water, a THz-TDS system coupled with a raster scanning imaging gantry produces intensity maps at 0.5 mm/pixel, which correspond to water distribution in a sample. In these maps the peak-to-peak amplitude of the pulse at each point determines the color of the pixel. To accomplish this, we used a Teraflash system with an imaging extension, both from Toptica. In our experiments we measured over a 70 ps window where two measurements were taken at each point and averaged. The thickness and material composition of a sample must be considered before measurement as thick samples or samples consisting primarily of highly conductive materials will not allow any radiation to pass through. For this investigation sample thickness could not exceed 6 mm and the minerals that compose the carbonate rocks, although somewhat absorptive, did not attenuate the signal excessively.

Porosity maps can be created using the varying levels of moisture in the samples by comparing 3 moisture maps of the same sample: (1) The sample with all pores saturated with water, (2) The sample with water removed from pores larger than a specific size, and (3) The sample with all water removed. The maps can then be compared using a custom image analysis script written for IgorPro. This script aligns the sample within each map, compares the intensity at each pixel, and generates the porosity maps. The map of the total porosity of the sample is generated by comparing maps 1 and 3; the map of the porosity smaller than a desired size is generated from maps 2 and 3, while the map of porosity larger than a desired size is generated from 1 and 2.

### Workflow

Samples were measured in sequence using the following procedure. The process was repeated two to three times per sample for the 1 µm diameter microporosity cut-off and performed only once for the 0.3 µm diameter microporosity cut-off. The dry sample was weighed to obtain the initial dry mass, *m*_*dry,i*_, and then submerged in the ion-saturated solution remaining from the pre-cleaning step to mitigate further calcium carbonate dissolution from the samples due to its finite solubility in water. The sample and fluid were placed under vacuum for 2 days. The saturated sample is removed from the vacuum chamber and fluid, excess water blotted, and weighed again to obtain the saturated mass, *m*_*sat*_, and a THz-TDS map (19 mm × 42 mm; 0.5 mm/pixel) was collected to map the water filling the fully saturated pore space.

The sample is then centrifuged using an Eppendorf 5804 with an FA-45–6-30 fixed angle rotor to the clear large pores based on the applied capillary pressure. Equation (1) relates pore radius, *r*_*p*_, contact angle, *θ*, and interfacial tension, *γ*, to the capillary pressure, *P*_*c*_, of a capillary tube.

1 $${P}_{c}=\frac{2\sigma {\cos}\theta }{{r}_{p}}$$

The samples were assumed to be water wet with a contact angle of 45° and the interfacial tension of 72.8 mN/m was used for air displacing water. With these properties, a *P*_*c*_ of 30 psi corresponds to a pore diameter, *d*_*p*_, cut-off of 1 µm and a *P*_*c*_ of 100 psi corresponds to a *d*_*p*_ cut-off of 0.3 µm. The rotational speed for the centrifuge was adjusted to reach the desired *P*_*c*_; 3140 rpm for 30 psi to clear pores with *d*_*p*_ > 1 µm and 5720 rpm for 100 psi to clear pores with *d*_*p*_ > 0.3 µm. Following centrifugation, the sample was weighed to obtain the centrifuged mass, *m*_*cent*_, and another THz-TDS spectroscopy map collected to map the remaining water in filling the micropores smaller than the *d*_*p*_ cut-off.

Finally, the samples are oven dried at 100 °C, weighed to obtain the final dry mass, *m*_*dry,f*_, and the final THz-TDS spectroscopy map collected.

The full workflow was repeated two to three times per 2 mm, 4 mm, and 6 mm samples at the *d*_*p*_ > 1 µm threshold and one time for the 4 mm and 6 mm samples at the *d*_*p*_ > 0.3 µm threshold. It should be noted that some of the samples fractured during centrifugation. When possible, the pieces were recovered from the centrifuge tube, weighed, and pieced back together for THz mapping.

#### Porosity and microporosity by mass

The mass data were used to calculate the total and microporosity. The total porosity (*ϕ*_*m,t*_) is calculated as the ratio of the saturated pore volume, *V*_*p*_, to the bulk volume, *V*_*b*_, of the sample as shown in Eq. (2)

2 $${\phi }_{m,t}=\frac{{V}_{p}}{{V}_{b}}$$

where *V*_*p*_ is calculated using the mass balance between the saturated rock, *m*_*sat*_, and dry rock, *m*_*dry*_, and the density of water, *ρ*_*w*_, as 1 g/cm^3^ as shown in Eq. (3).

3 $${V}_{p}=\frac{{m}_{sat}-{m}_{dry,i}}{{\rho }_{w}}$$

The bulk density, *ρ*_*b*_, of each sample is calculated from the mass and dimensions of each plug. The bulk density of the Samples 1 and 2 core plugs was 2.2 g/cm^3^ and the Sample 3 core plug was 2.3 g/cm^3^. The bulk volume, *V*_*b*_, is then calculated as *V*_*b*_ = *m*_*dry*_/*ρ*_*b*_.

The amount of microporosity (*ϕ*_*m,µpores*_) is determined following Eq. (2) except that the volume of micropores, *V*_*µpores*_, replaces the volume of total pores, *V*_*p*_. The volume of micropores, *V*_*µpores*_, is calculated from the mass balance using the mass of the rock after centrifugation, *m*_*cent*_, and the final dry mass of the rock, *m*_*dry,f*_, following the same form as Eq. (3). Finally, the relative amount of microporosity, *ϕ*_*m,µpores*_, to total porosity, *ϕ*_*m,﻿t*_, is calculated.

#### Microporosity by THz

While the goal of the THz imaging is not to directly calculate microporosity but to provide maps of the spatial variations in the microporosity within the sample, the sensitivity of the measurement to the water in the sample does provide a means to estimate the microporosity (*ϕ*_*THz,µpores*_) as shown in Eq. (4)

4 $${\phi }_{THz,\mu pores}=avg\left(\frac{\left(\frac{{THz}_{cent,ij}}{{THz}_{sat,ij}}\right)}{max\left(\frac{{THz}_{cent,ij}}{{THz}_{sat, ij}}\right)}\right)*avg\left(\frac{\left(\frac{{THz}_{sat,ij}}{{THz}_{dry,ij}}\right)}{{\rho }_{b} * max\left(\frac{{THz}_{sat,ij}}{{THz}_{dry,ij}}\right)}\right)$$

where *THz*_*cent,ij*_, *THz*_*sat,ij*_, and *THz*_*dry,ij*_ are the THz attenuation map data at each *ij* pixel within a region of interest (ROI) selected around the rock boundary. The maximum of the ratio of the two maps was used to account for power fluctuations that might occur due to fluctuations in the humidity of the air and changes in alignment. The relative amount of the relative amount of microporosity, *ϕ*_*THz,µpores*_, to total porosity from mass, *ϕ*_*t*_ is the calculated for comparison to mass balance and MICP.

### Mercury injection capillary pressure (MICP)

Remaining portions of the plugs sampled for THz imaging were used for MICP analysis (MetaRock Laboratories, Houston, TX). A full description of the MICP technique and apparatus can be found in general petrophysics textbooks^13^. Plug samples were tested in the as-received state and then dried in an oven at 100 °C for 24 h to remove water. The sample is then placed in the sample holder and mercury is applied to determine the bulk volume of the sample at atmospheric pressure. The rock is then subjected to increasing pressures at pre-determined steps and timepoints where the volume of mercury injected into the sample is measured versus applied pressure. At each step the applied pressure overcomes the capillary pressure threshold barrier to fill pores of a specific size as estimated by the capillary pressure equation (Eq. 1). Each pressure is converted to a pore size and the volume of mercury injected at each pressure corresponds to the volume of pores of the pressure-designated pore size to generate a pore size distribution (PSD). The total volume of mercury injected relative to the bulk volume of the sample provides the MICP porosity. The maximum pressure applied in MICP is 60,000 psi corresponding to a pore diameter of 3 nm. The microporosity from MICP are obtained from the cumulative pore size distribution (PSD) curve.

### Micro X-ray fluorescence (micro-XRF)

Elemental maps at 20 µm/pixel were collected with a M4 Tornado micro-XRF (Bruker, USA) of each sample to show variations in the rock mineralogy and texture. The samples were placed under vacuum and irradiated at 50 kV with a current of 200 µA.

### X-ray computed tomography (CT)

X-ray computed tomography (CT) scans were collected using a NSI X5000 Industrial CT scanner (North Star Imaging, Inc.) to observe the grain and large pores distributions within the THz samples and the remaining portion of the plugs used for MICP. The scans were performed at 720 views per rotation where the reconstructed voxel size was 50 µm × 50 µm × 50 µm. The CT radiographs were reconstructed with the iTomoFDK software (iTomography, Houston, TX) and three-dimensional visualizations of the three cement samples were created using GeoDict (Math2Market). ImageJ was used to visualize and crop images.

## Supplementary Information

Supplementary Figures.

## Data Availability

The data sets generated during and/or analyzed during the current study are not publicly available due to the corresponding author’s corporate affiliation but are available from the corresponding author on reasonable request.
