# Supplementary material for: Terahertz imaging for non-destructive porosity measurements of carbonate rocks
Source: Sci Rep. 2022 Oct 26;12:18018. doi: 10.1038/s41598-022-22535-z (PMC9606024; doi:10.1038/s41598-022-22535-z)
Supplement: Supplementary file 1 — Supplementary Figures. [file 41598_2022_22535_MOESM1_ESM.docx]

**Supplemental Information**

**Terahertz imaging for non-destructive porosity measurements of carbonate rocks**

**Jacob Bouchard^1*^, Shannon Eichmann^2*^, Hooisweng Ow^3^, Martin Poitzsch^3^, Douglas T Petkie^1^**

### Affiliations

**^1^Department of Physics, Worcester Polytechnic Institute, Worcester, MA, USA**

**^2^Aramco Americas: Aramco Research Center – Houston, Houston, TX, USA**

**^3^Aramco Americas: Aramco Research Center – Boston, Cambridge, MA, USA**

***Contributed equally to this work.**


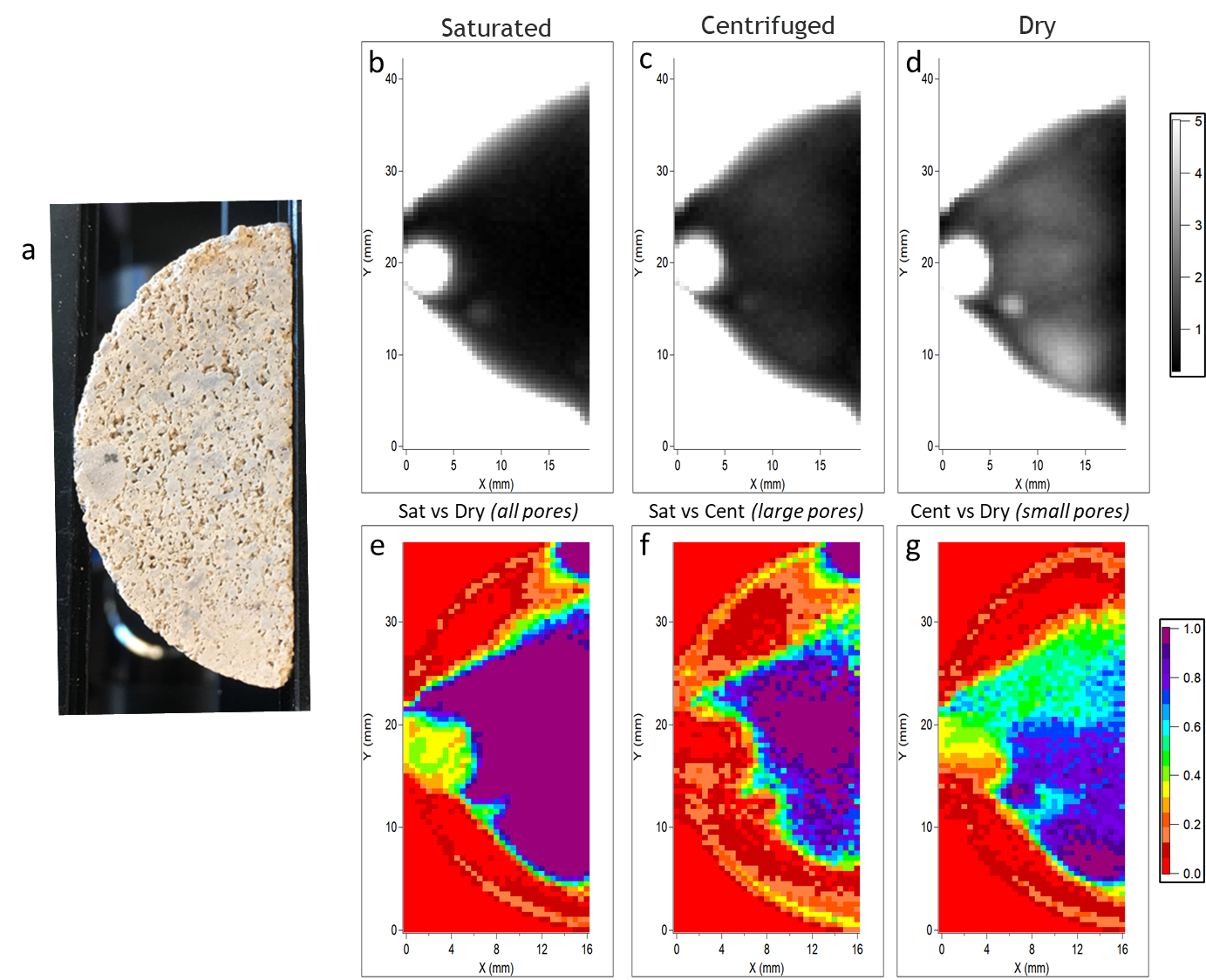


**Figure S1. Attenuation and attenuation difference THz maps of Sample 1 (4 mm thick) as representative examples of the data for all samples.** (a) photo, (b-d) normalized attenuation maps of the saturated, centrifuged, and dry samples, and (e-g) difference maps between the saturated and dry, saturated and centrifuged, and centrifuged and dry attenuation maps to represent the spatial variation of the saturated pores, macropores (*d_p_* > 1 µm), and micropores (*d_p_* < 1 µm).

**
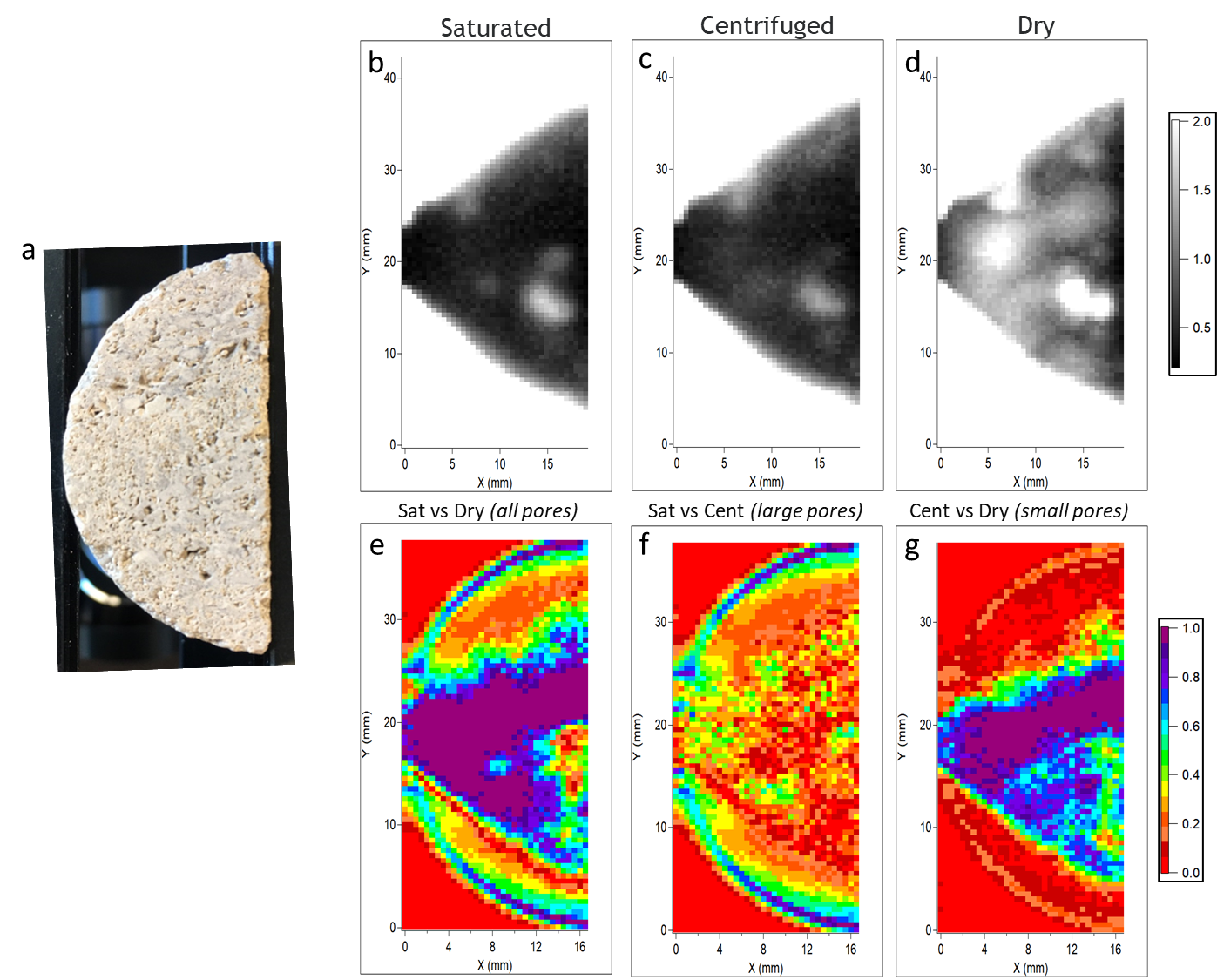
**

**Figure S2. Attenuation and attenuation difference THz maps of Sample 2 (4 mm thick) as representative examples of the data for all samples.** (a) photo, (b-d) normalized attenuation maps of the saturated, centrifuged, and dry samples, and (e-g) difference maps between the saturated and dry, saturated and centrifuged, and centrifuged and dry attenuation maps to represent the spatial variation of the saturated pores, macropores (*d_p_* > 1 µm), and micropores (*d_p_* < 1 µm).

**Figure S3 micro-XRF Maps to be added**

**Figure S3. micro-XRF Maps for 4mm thick samples (face showing in photos accompanying Figs. 3, S1, and S2).** Elemental maps of calcium (Ca), magnesium (Mg), iron (Fe), sulfur (S), chlorine (Cl), and aluminum (Al) are shown as these highlight variations between the samples. All showing Ca is dominant but Sample 1 shows distributed Mg patches while Samples 2 and 3 show more Fe, S, Cl, and Al distributed. Images shown are false colored and scaled to show variations. Intensities of the images are not quantitative, bright pixels outside of sample area are caused by rescaling or sample holder.
